# Discussion of Cuffless Blood Pressure Prediction Using Plethysmograph Based on a Longitudinal Experiment: Is the Individual Model Necessary?

**DOI:** 10.3390/life12010011

**Published:** 2021-12-22

**Authors:** Koshiro Kido, Zheng Chen, Ming Huang, Toshiyo Tamura, Wei Chen, Naoaki Ono, Masachika Takeuchi, Md. Altaf-Ul-Amin, Shigehiko Kanaya

**Affiliations:** 1 Graduate School of Science and Technology, Nara Institute of Science and Technology, Ikoma 630-0192, Japan; kido.koshiro.kb3@is.naist.jp (K.K.); chen.zheng.bn1@is.naist.jp (Z.C.); nono@is.naist.jp (N.O.); amin-m@is.naist.jp (M.A.-U.-A.); skanaya@gtc.naist.jp (S.K.); 2 Institute for Healthcare Robotics, Waseda University, Tokyo 162-0041, Japan; t.tamura3@kurenai.waseda.jp; 3 Department of Electronic Engineering, School of Information Science and Technology, Fudan University, Shanghai 201203, China; w_chen@fudan.edu.cn; 4 Data Science Center, Nara Institute of Science and Technology, Ikoma 630-0192, Japan; 5 San-Ei Medisys Co., Ltd., Kyoto 607-8116, Japan; takeuchi@san-ei.com

**Keywords:** blood pressure, cuffless measurement, longitudinal experiment, plethysmograph, nonlinear regression

## Abstract

Using the Plethysmograph (PPG) signal to estimate blood pressure (BP) is attractive given the convenience and possibility of continuous measurement. However, due to the personal differences and the insufficiency of data, the dilemma between the accuracy for a small dataset and the robustness as a general method remains. To this end, we scrutinized the whole pipeline from the feature selection to regression model construction based on a one-month experiment with 11 subjects. By constructing the explanatory features consisting of five general PPG waveform features that do not require the identification of dicrotic notch and diastolic peak and the heart rate, three regression models, which are partial least square, local weighted partial least square, and Gaussian Process model, were built to reflect the underlying assumption about the nature of the fitting problem. By comparing the regression models, it can be confirmed that an individual Gaussian Process model attains the best results with 5.1 mmHg and 4.6 mmHg mean absolute error for SBP and DBP and 6.2 mmHg and 5.4 mmHg standard deviation for SBP and DBP. Moreover, the results of the individual models are significantly better than the generalized model built with the data of all subjects.

## 1. Introduction

Ambulatory blood pressure (BP) monitoring provides abundant cardiovascular information, and we have seen numerous studies focusing on replacing the conventional auscultatory/oscillometric measurement that requires the occlusion of arterial blood flow cuffless by using the cardiovascular biosignals with state-of-the-art machine learning methods.

The cuffless methods can be roughly categorized into three groups. The first one is based on the pulse arrival time (PAT) [1,2], the second one is based on photoplethysmograph (PPG) signal, which has attracted more and more attention in recent years [3,4,5,6], and the third one is based on other methods [7,8].

The PAT is the period that includes the pulse transition time and the pre-ejection period of the heart. Because the PAT needs the ECG signal and distal PPG signal to be determined, it is a modality that is suitable for intermittent measurement.

The second group utilizes only one biosignal—the PPG, which is a signal that reflects the change of blood volume. The pulsatile component of PPG reflects the change in blood volume and the stable component is related to the basic blood volume and other physiological indices such as respiration and body temperature [9]. Since the PPG signal depends on the blood volume in its optical path, which typically covers the arterial and venous capillaries [10], it can be related to the cardiovascular indices such as the blood oxygen saturation and arterial compliance [11,12]. It is generally accepted that the PPG waveform comprises the arterial pulse wave from the left ventricle to the distal sites and the reflected wave from the sites of impedance mismatch [13]. With these physiological understandings, research interest has been shifting to BP estimation based on PPG signal recently. Xing et al. carried out a mass experiment (1249 subjects, 2358 measurements), and by using the PPG waveform features and biometrics in a bagged regression tree model, they got a 9.5 mmHg, 2.2 mmHg, and 17.4 mmHg mean absolute error (MAE) for hypotensive, normotensive, and hypertensive subjects [3]. Chowdhury et al. used an open dataset obtained in a hospital with 219 subjects and 657 measurements to extract the PPG waveform features and biometrics, which were then inputted into the Gaussian Process Regression model. By using a further Bayesian optimization, they claimed 3.02 mmHg and 1.74 mmHg MAE for SBP and DBP estimation, respectively [6].

There are two main criteria for the cuffless method. The first one is that the method/system should meet the requirement on accuracy determined by IEEE Std 1708-2014. In the mean time, when used as a healthcare device, the convenience for personal use should also be taken into account. Therefore, the final goal of development should be an appropriate computation model with high accuracy and long-term stability.

These preliminary studies show the prospect of cuffless BP estimation with PPG signal; however, fundamental issues remain. The first one is the dilemma between the generalization of the regression model and the individual difference of the limited data. This kind of difference is pervasive in biomedical engineering and the method of making a balance is reflected in the modeling assumption and the choice of machine learning model. Data-driven approach resorts to the accumulation of samples to find the relation between explanatory and dependent variables. A globally nonlinear regression model that severely twists the fitting hyperplane to fit the data on hand may not be well applicable for an individual outside the dataset, especially when the size of the dataset is small. It is not uncommon to see the difficulty in getting a reproducible relation between the waveform features and the blood pressure learned from a small dataset in a large population test. The relation may be altered substantially by the demographic factors and physiological/pathological factors. For example, Allen has confirmed the PPG contour triangulation with aging [14]. This means that a robust method should not rely on the identification of dicrotic notch and/or diastolic peak in the PPG waveform.

Moreover, the cardiovascular functions including BP are regularized by biorhythms modulated by the baroreflex and the autonomic nervous system [15,16], which implies the necessity of re-calibration for long-term use.

Based on the discussion above, a through discussion is necessary before the large scale validation experiment. In this study, we tried to contribute to this topic by answering the following two fundamental questions: (*Q1*) When compared with the individual model, is the generalized model good enough? (*Q2*) Is it necessary to calibrate in a relatively long-term scenario? Special attention is also paid to the following aspects of lift performance (corresponding to *A1*) and robustness (corresponding to *A2*) of the model: (*A1*) to decide the best model for BP regression by comparing the linear, local linear, and Bayesian models; (*A2*) to examine the feasibility of using the general waveform features solely in the regression models, which means that the identification of the dicrotic notch and diastolic peak is unnecessary to address the deterioration of the dicrotic notch and the diastolic peak with aging and hypertension.

## 2. Materials and Methods

To the best knowledge of the authors, there are very few open datasets dedicated to the study of cuffless BP estimation. For example, the MIMIC database is well used in blood pressure estimation, whose protocol is not optimized for the BP study. The Non-invasive Blood Pressure Estimation dataset [17], collected data right after running, which would change the behavior of the baroreflex [18].

In view of the situation that none of the open dataset can be used to answer the questions Q1 and Q2, a one-month experiment, from mid-May to mid-June, dedicated to the development and validation of cuffless BP measurement was conducted. The experiment used a PPG-sensor with 940 nm LED (infrared) of a medical device (Checkme Pro B, San-ei Medisys) for 30 s PPG measurement and a clinical Blood Pressure Monitor (A&D, Model UA-1200BLE) as the reference [19]. Eleven subjects, whose basic information can be found in Table 1, participated in the experiment, during which they were required to measure the PPG signal and BP values multiple times (typically 4 times/day) a day in different periods. Subjects were guided to rest for three minutes before taking the measurements in a sitting position. The study was approved by the Ethics Review Committee of the San-ei Medisys Company (#2019002SA). All methods were performed in accordance with the relevant guidelines and regulations. Informed consent was obtained from all the subjects.

### 2.1. Preprocessing

This research differentiates itself from the previous studies that tried to extract the morphological features of PPG waveforms as many as possible and then used the features selection methods to pick up the important ones to input into the regression models. We argue that the whole pipeline should take serious account of the problem that the deterioration of the dicrotic notch and the diastolic peak with aging and hypertension will bring uncertainty to the relevant features. This is also the reason why we highlight the *A2* aspect in our study.

On this account, the explanatory features set X consists of (1) the heart rate reading (*HR*) during the experiment; and (2) the general PPG waveform features, which are described as follows. Firstly, 6th order Butterworth IIR low pass zero-phase filter ($f_{c}$ = 10 Hz) and a linear detrend process were applied to the PPG signals before the following procedure:

Firstly, the skewness values, which is a well-used index for the signal quality index (SQI) of PPG signal [20], of beat-by-beat PPG waveforms in each sample was calculated and the waveform with the maximum skewness values was picked, thereafter the sample with the maximum SQI less than 0.1 will be removed.

**Figure 1:**
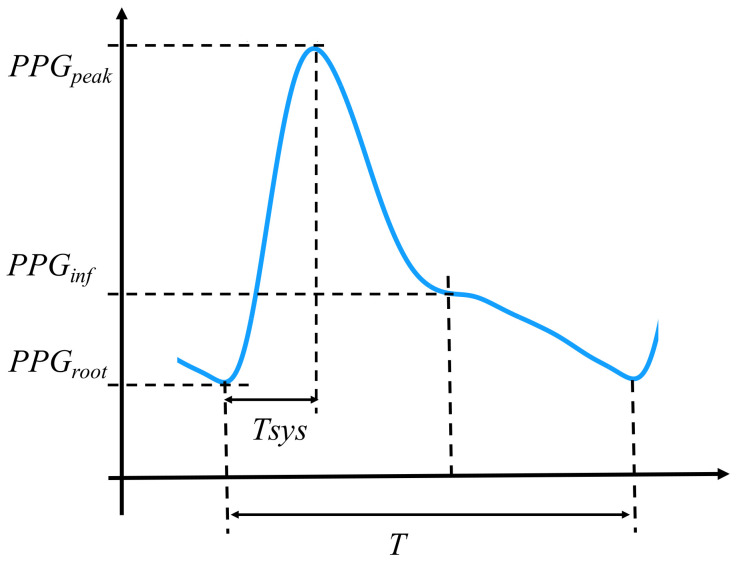
Illustration of PPG features. Inflection point of the PPG waveform between the dicrotic notch and the diastolic peak is used given that the disappearance of the diastolic peak may appear.

Secondly, the following features (Figure 1) of the waveform were calculated:

- *PPG* intensive ratio (*PIR*): $PIR=PPG_{peak}/PPG_{root}$, which is an index to reflect changes in the arterial diameter [21];
- Diastole time ratio (*DTR*): $DTR=t_{sys}/T$, where the $t_{sys}$ is the time from the systole peak to the end of a PPG waveform, and *T* is the time length of the corresponding *PPG* waveform;
- *ri*: $ri=PPG_{peak}/PPG_{inf}$, where the $PPG_{inf}$ is the inflection point of the PPG waveform between the dicrotic notch and the diastolic peak. This parameter is introduced given the disappearance of the diastolic peak, while the inflection point can be found. Given the fact that the PPG amplitudes of inflection point and the diastolic peak are similar, the *ri* is used as the alternative of the Reflection index [22];
- *A02*: Area of the 0–2 Hz band of the PPG waveform [6];
- *A25*: Area of the 2–5 Hz band of the PPG waveform [6].

The five features above, which have been validated in the previous studies [4,6,21], were selected based on the underlying consideration that potential PPG features should avoid the identification of the dicrotic north and the diastole peak. Meanwhile, the HR, which has been proved to be informative in BP estimation [16,23], is used as another explanatory variable.

### 2.2. Regression Models

To respond to the *A1*, three regression models were used in this research. While the PLS corresponds to the linear assumption of the features (X) and the BP (y), the LWPLS corresponds to the assumption of local linearity defined by the close samples in the features space. The Gaussian Process Regression can be viewed as a nonlinear regression by using a Radial Basis kernel.

#### 2.2.1. Partial Least Squares (PLS)

By projecting the input into new spaces, PLS can handle the problem with visible collinearity in the explanatory variables. In a problem with $X\in {ℜ}^{N\times P}$ as the explanatory variables (*N*: the number of samples; *P* the number of the explanatory variables) and $y\in {ℜ}^{N}$ as the dependent variable, the normalized X and y are expressed alternatively as follows:

(1) $$X=TP^{T}+E,$$

(2) y=Tq+f,

where the $T\in {ℜ}^{N\times D}$ is the matrix that contains latent components in each column, and $P\in {ℜ}^{P\times D}$ is the matrix that contains the loading of each variable for the components in each row. $q\in {ℜ}^{D}$ is the regression coefficient vector from latent variables to the output. E and f are the residuals.

The projection of X and y is done iteratively by maximizing the covariance of the $y^{T}t_{i}$, where $t_{i}$ is the *i*th column of the T, taking the first two latent components as an example.

(3) $$t_{1}=Xw_{1},$$

where the $w_{1}$ is the weight vector for the first component with the regularization that $∥w_{1}∥=1$. By using the *Largrange* multiplier and least squares method, the $w_{1}$, the $t_{1}$, and the corresponding $p_{1}$ and $q_{1}$, which is the first row of P and the first element of q respectively, can be calculated. Similarly, the second component can be derived by subtracting the projection in the first component from the X and y,

(4) $$X_{2}=X-t_{1}p_{1}^{T},$$

(5) $$y_{2}=y-t_{1}q_{1},$$

and calculate the parameters by

(6) $$w_{2}=\frac{X_{2}^{T}y_{2}}{∥X_{2}^{T}y_{2}∥},$$

(7) $$P_{2}=\frac{X_{2}^{T}t_{2}}{t_{2}^{T}t_{2}},$$

(8) $$q_{2}=\frac{y_{2}^{T}t_{2}}{t_{2}^{T}t_{2}}.$$

The procedure is repeated when the *i* reaches the number *D*, whose value can be determined by using the cross-validated coefficient of determination ($R_{cv}^{2}$).

(9) $$r_{cv}^{2}=1-\frac{\sum_{i=1}^{N}{\left(y^{i}-y_{cv}^{i}\right)}^{2}}{\sum_{i=1}^{N}{\left(y^{i}-y_{A}\right)}^{2}},$$

where the $y^{i}$ and the $y_{CV}^{i}$ are the true value and the estimate of the *i*th sample in the cross validation, respectively; the $y_{A}$ is the mean of y. The *i* is set as 2 after the confirmation with $r_{cv}^{2}$.

#### 2.2.2. LW-PLS

As a just-in-time method, the LW-PLS is conceptually different from the PLS. It separates the samples to a training dataset with $X_{t}\in {ℜ}^{M\times P}$ and $y_{t}\in {ℜ}^{M}$ and the new request $x_{r}\in {ℜ}^{P}$, whose similarity will be compared with the training dataset and used to generate the diagonal similarity matrix.

(10) $$U=\left[\begin{array}{ccc} u_{r}^{1} & \cdots & 0\\ \vdots & \ddots & \vdots \\ 0 & \cdots & u_{r}^{M} \end{array}\right]$$

where the $u_{q}^{i}$ is the similarity between the *i*th sample in $X_{t}$ and the $x_{r}$. The $X_{t}$ and $y_{t}$ are adjusted based on the U.

(11) $$X_{0}=X_{t}-X_{w},$$

(12) $$y_{0}=y_{t}-y_{w}.$$

The $X_{w}$ and $y_{w}$ are generated as follows.

(13) $$X_{w}=\left[\begin{array}{c} 1\\ 1\\ \cdots \\ 1 \end{array}\right]\left[\begin{array}{c} x_{w,1},x_{w,2},\cdots ,x_{w,P} \end{array}\right],$$

and

(14) $$y_{w}=\frac{\sum_{i=1}^{M}u_{r}^{i}y^{i}}{\sum_{i=1}^{N}u_{r}^{i}},$$

where,

(15) $$x_{w,j}=\frac{\sum_{i=1}^{M}u_{r}^{i}x_{j}^{i}}{\sum_{i=1}^{N}u_{r}^{i}}.$$

With all this preparation the first component can be calculated.

(16) $$w_{1}=\frac{X_{0}^{T}Uy_{0}}{∥X_{0}^{T}Uy_{0}∥},$$

(17) $$t_{1}=X_{0}w_{1},$$

(18) $$p_{1}=\frac{X_{0}^{T}Ut_{1}}{t_{1}^{T}Ut_{1}},$$

(19) $$q_{1}=\frac{y_{0}^{T}Ut_{1}}{t_{1}^{T}Ut_{1}},$$

The $p_{1}$ and $q_{1}$ represent the loading vector and coefficient of the first component, respectively. The parameters are determined iteratively when the predefined component number is met. The estimate for the request $x_{r}$ is the summation of the ${\hat{y}}_{a}=\sum_{i=1}^{D}t_{a,i}q_{a,i}+y_{w}$.

The distance index is crucial to the LW-PLS. Therefore, special care should be paid to its choice. In this research, it is considered that the *Mahalanobis* distance, which is the improved *Euclidian* distance to amend the problems of different scales and correlation between explanatory features with the definition below

(20) $$d_{m}=\sqrt{{\left(x_{i}-x_{j}\right)}^{T}S^{-1}\left(x_{i}-x_{j}\right)},\left(x_{i}\;\mathrm{and}\;x_{j}:\;\mathrm{the}\;\mathrm{samples};\;S:\;\mathrm{covariance}\;\mathrm{matrix}\right)$$

is suitable for this heterogeneous set of explanatory features consisting of continuous ones of different scales.

#### 2.2.3. Gaussian Process Regression

Gaussian Process Regression is fundamentally a kernel method, which defines the similarity of explanatory variables in terms of a kernel function. Instead of the explicit definition of the basic functions $\phi_{i}\left(x\right),\left(i=1,2,\ldots ,l\right)$ and determination of the optimized weights vector w, where $y\left(x,w\right)=w^{T}\phi \left(x\right)$, the Gaussian Process defines a prior probability distribution over the basic functions. For a dataset with *N* samples, the equation becomes

(21) $$y=\Phi w,\mathrm{where}\;\Phi \;\mathrm{is}\;\mathrm{the}\;\mathrm{design}\;\mathrm{matrix},\;\Phi_{nk}=\phi_{k}\left(x_{n}\right).$$

By letting the prior distribution of the weights vector to be Gaussian, $p\left(w\right)∼N\left(w|0,\alpha^{-1}I\right)$ and then defining the kernel function $k(x,x^{\prime })$, where

(22) $$k\left(x,x^{\prime }\right)=\frac{1}{\alpha }\phi {\left(x\right)}^{T}\phi \left(x^{\prime }\right),$$

The Gaussian Process can be defined by the kernel function, since E[y]=ΦE[w]=0

(23) $$E\left(y\left(x_{n}\right)y\left(x_{m}\right)\right)=k\left(x_{n},x_{m}\right),$$

In this research, the kernel is the Radial Basis Function (scale = 1) contaminated with white noise (noise level = 1) for the standardized explanatory variables.

### 2.3. Calibration Schemes

The personality is reflected by incorporating the personal samples into the training dataset. Two kinds of calibration schemes were used. The first scheme (scheme_1) used the first 20% (typically 23 samples) of the samples in the training process; whereas the second scheme (scheme_2) used the first 4% and the 15th–18th, 35th–38th, 55th–58th, 75th–78th, and 95th–98th in the training process, which were roughly 20% of the individual samples as well. Of note, to prevent the leaking of future information, the BP values are predicted by using the previous calibration readings. That is, for example, the 19th-34th BP values are predicted by using the initial and the 15–18th calibration readings. These two schemes were used to examine the necessity of re-calibration in a relatively long-term scenario (*Q2*). Specifically, if the best results come from the the scheme_2, it is reasonable to conclude that the model should take the biorhythm, such as seasonality, into account and the re-calibration should be considered.

### 2.4. Generalized and Individual Models

To answer the question *Q1*, two training strategies were used in model construction. A generalized model trains the model as a generalized one by using the individual calibration samples as well as the samples from the other subjects. The other strategy is to construct an individual model, which was trained by using individual calibration samples solely. The two calibration schemes above were used in both models. The mean absolute error (MAE), as well as the mean and standard deviation of the regression error, were used as the main metrics; particularly, the MAE was used to determine the best model.

## 3. Results

By summarizing the one-month experiment, all of the 1287 samples collected from the 11 subjects, whose information is shown in Table 1, were used in the following modeling. The numbers of samples for the subjects are even, from 114 to 118.

### 3.1. Results of Generalized Models

**Figure 2:**
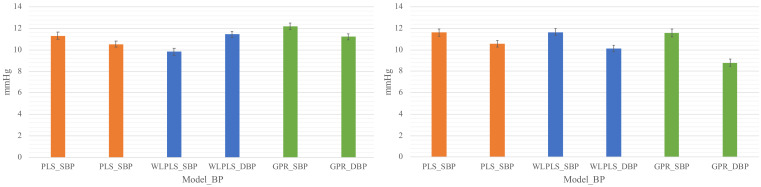
The mean absolute error (MAE) of systolic and diastolic BP of the three generalized models. The bar plot on the left corresponds to the results with scheme_1 and the bar plot on the right to the results with scheme_2. Sticks show the standard errors.

In this study, the linear partial least square model (PLS), the local linear local-weighted partial least square model (LWPLS), and the Gaussian Process Regression model (GPR) were used to reflect different assumptions about this regression problem. The generalized model is trained in the manner of leave-one-out validation, by taking in all samples in the training dataset (10 subjects) merging the individual calibration samples (calibration scheme_1 and scheme_2, more detail in **Methods**). By summarizing the regression results of the testing samples, the mean absolute error (MAE) of systolic and diastolic BP is shown in Figure 2, from which it can be seen that none of the models can fit the personal well with MAE larger than 9 mmHg. Therefore, no further comparison of features was conducted for the generalized models.

### 3.2. Comparison of Different Individual Models

**Figure 3:**
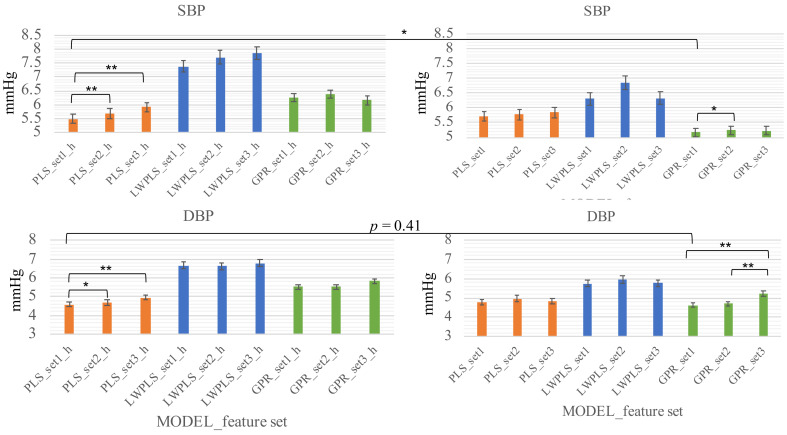
Comparison of three individual models using scheme_1 (left column) and scheme_2 (right column). Sticks show the standard errors.The setx means the combination of PPG features, where the set1 is {*hr*, *a02*, *a25*, *ri*}, set2 is {*pir*, *dtr*, *hr*, *a02*, *a25*, *ri*}, set3 is {*hr*, *ri*}. * means the *p* < 0.05 and, ** means the *p* < 0.001. setx_h corresponds to the scheme_1 calibration.

Individual models were constructed by taking the calibration samples from the same subject for model construction and testing the samples remaining, whose results are summarized in Figure 3. As introduced in the **Methods** section, two calibration schemes were used. The left column corresponds to the calibration using the initial 20% samples (scheme_1), while the right column corresponds to the situation of re-calibrating every 20 samples (scheme_2).

By comparison with the generalized models, each model significantly outperforms its counterpart (*p* < 0.001). This suggests that the individual model is more appropriate in building the BP regression model in the current stage.

The three models with two calibration schemes were examined by altering the combination of explanatory features. Therefore, there are three trials for each model, as shown in Figure 3. Inside each scheme, we only compare the model having the lowest MAE with others that have close MAEs by pair-wise student *t*-test and the models with lowest MAE from each scheme. For SBP, the best models are PLS_set1 for shceme_1 and GPR_set1 for scheme_2. Moreover, the GPR_set1 (scheme_2) is significantly smaller than PLS_set1 (shceme_1) and smaller than GPR_set2 (scheme_2); it is considered as the best model for SBP.

For DBP, being consistent with the situation of SBP, the best models are PLS_set1 (shceme_1) and GPR_set1 (scheme_2). However, no significant difference can be found from these two models (*p* = 0.41). Of note, regarding the individual model that uses the scheme_2 calibration, linear interpolation that used two consecutive calibration windows has also been conducted to confirm the advantage of nonlinear regression using PPG features. For example, the averaged BP values of the two calibration windows of the 15th–18th samples and the 35th–38th samples are used to interpolate the 19th–34th BP values. The MAEs of SBP and DBP are 5.96 and 4.72 mmHg, respectively. The accuracy of SBP is significantly lower than GRP_set1; whereas no significant difference can be seen in DBP. Combining these pieces of result, the feature combination set 1 is consistently the best. Moreover, the GPR coupled with the re-calibration scheme (scheme_2) has a significantly low MAE for SBP (5.16 mmHg) and similar MAE for DBP (4.63 mmHg); it is considered as the best method for BP estimation.

### 3.3. Regression Model

**Figure 4:**
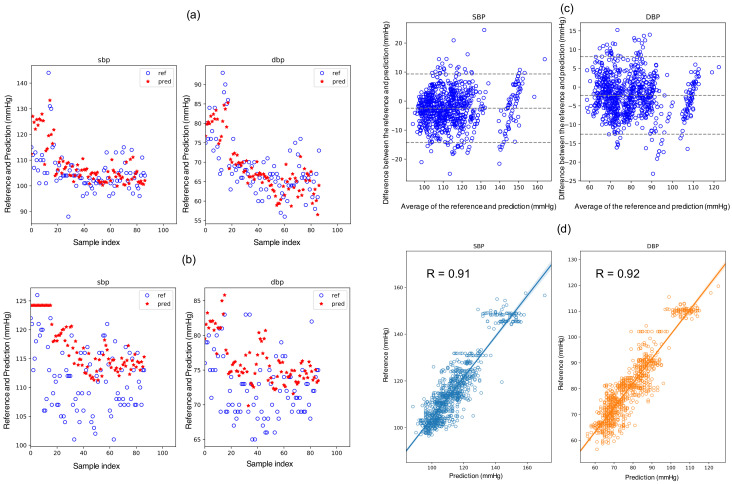
The results of the GPR_set1 (scheme_2) model. The fitting of the one-month samples of two randomly selected subjects can be seen in (**a**,**b**). The BA plot for and linear correlation can be found in (**c**,**d**).

Based on the results above, the GPR model with explanatory features of *hr*, *a02*, *a25*, and *ri* is used for both SBP and DBP regression. The results of the regression model are shown in Figure 4, from which it can be seen that the overall trend of the BP can be captured (Figure 4a,b). The decreasing trend of both the SBP and DBP may be attributed to the seasonal influence and can be observed from most of the subjects [24,25]. The mean and standard deviation of the fitting are

- SBP: −2.2 ± 6.2 mmHg; DBP: −2.0 ± 5.4 mmHg.

The individual fitting results are tabulated in Table 1, from which it can be confirmed that most of the subjects have SBP MAE values less than 6 mmHg, except for No. 3, 4, and 11, whose BP ranges were relatively wide.

## 4. Discussion

The observation that the generalized models are significantly inferior to individual models is consistent with the finding of Gašper et al. [26] based on the MIMIC data. This reconfirmation suggests that the samples (features and BP values) from different individuals follow different distributions. It also suggests that the generalizability of a BP model should be validated on a dataset within a certain period.

**Figure 5:**
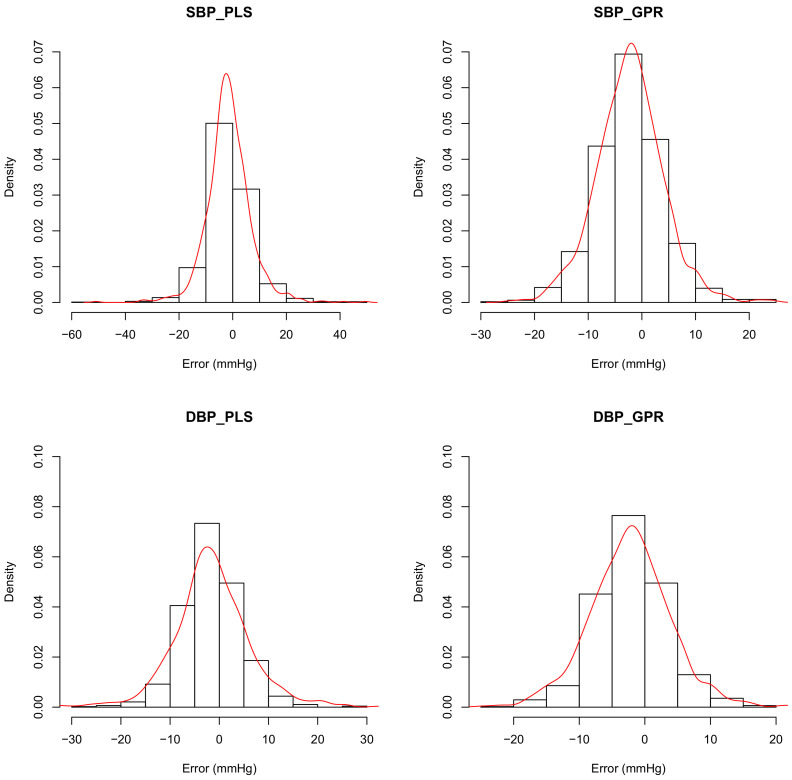
Distribution of predicting errors of the PLS_set1 (**left** column) adn GPR_set1 models (**right** column). Red curves show the approximation of probability density using Gaussian distribution.

Although the ISO81060-2 uses the mean as a major criterion in model evaluation, MSE, which is more sensitive to the variance of the error, is often used in model selection [6,23]. In this study, the Mean errors of SBP and DBP with the PLS_set1 model are −1.46 and −1.53 mmHg, respectively. However, the correlation values are evidently lower as 0.82 and 0.89, respectively. Figure 5 shows the distributions of the predicting errors of PLS_set1 (left column) and GPR_set1 (right column). It can be confirmed in Figure 5 that the bias values of the PLS_set1 model are less; however, variances are higher than the GPR_set1 model. The MAE also stands out in its insensitivity to the outliers compared with the mean square error, which is used pervasively in machine learning. Therefore, the MAE is used in model comparison in this study.

The fact that the re-calibration scheme (scheme_2) surpasses the initial-calibration scheme (scheme_1) in systolic BP suggests that the model may only capture the fluctuation of the systolic BP within a couple of days and there is a component, periodically or not, with a longer period in the systolic BP change. On the other hand, the two schemes showed little difference in diastolic BP (lower row of Figure 4). This observation suggests that the diastolic BP is more stable than the systolic BP.

The necessary number of samples in a calibration window and the length of calibration-free interval between windows are important in planning a subsequent experiment with a larger population and longer duration. By setting the target accuracy as 5 mmHg for both SBP and DBP, other settings of the scheme_2 have been tried, for example, two times of re-calibration at the 30th–40th, and the 70–80th samples result in significantly lower accuracy (MAE: 6.28 and 5.02 mmHg for SBP and DBP, respectively). Given that re-calibration too often may cause stress, an interval of 17–20 samples (4 to 5 days typically), and four samples of a calibration window (1 day typically), is suitable.

Another advantage of the re-calibration scheme is the swift initialization, which requires five measurements typically, whereas the initial-calibration scheme requires a long period, 23 measurements typically, for initialization to have acceptable accuracy. Please note that the granularity of this conclusion is the number of days based on the setting of the experiments. Whether repeating the calibrating measurements in a shorter interval, e.g., several minutes, can get similar accuracy could be validated subsequently.

**Figure 6:**
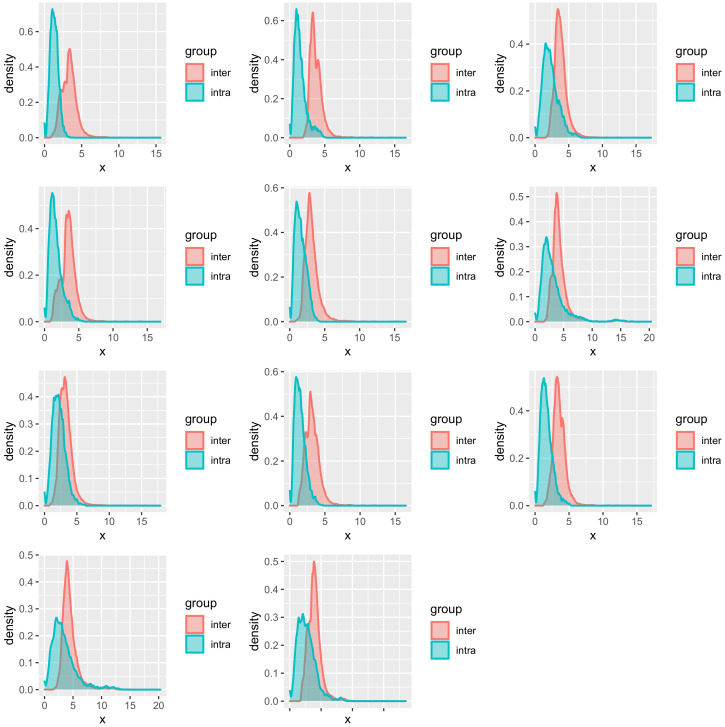
Distribution of similarity. Each subfigure corresponds to one subject. In each subfigure, the histogram in red shows the distribution of similarity of samples from the same subject; whereas the one in blue shows the distribution of similarity with other subjects.

The necessity of an individual model could be explained partially by further plotting the distribution of similarity based on *Mahalanobis* distance (Figure 6). The intra-similarity of the explanatory variables from the same subject is higher than the inter-similarity but with partial overlapping. Since in kernel-based regression, the LWPLS and the GPR will take close samples in the explanatory variables space to predict the new sample, the overlapping samples may be detrimental for the prediction by deviating the predicting hyperplane.

Regarding the choice of the regression model, this insight is especially significant in the era of deep learning, which is fundamentally the data-driven approach. A shallow network model can give an accurate estimation for the dataset on hand given the extraordinary capability in twisting the hyperplane to fit the samples. However, given the strong personality underlying this problem, a few-shot learning scheme that relates the same PPG and relevant features from the same subject with the change of BP fluctuation may be a method worth trying at the present stage. Although the application of the fully data-driven approach, e.g., the end-to-end deep learning pipeline, onto this topic is still difficult for the lack of individual longitudinal data, the information hidden in the sequence of EEG waveforms could be used by recurrent neural network or attention structure in the near future with further accumulation of data. In this regard, the construction of long term individual datasets should be the next key work.

An interesting study that significantly increases the training samples by extending the measurement duration (30 min) and then uses the BiLSTM model to predict the BP values hints that the deep learning structures should be carefully used for this topic. The main conclusion of the leave-one-subject-out validation that the model pretrained with population samples and fine-tuned with individual data attains the best performance may be partially attributed to a more informative feature space constructed by ECG, PPG, and BCG signal than using the PPG sensor solely. That is, the general relation between the features extracted from multiple sensors and the BP values could be captured and used in transfer learning. Although the physiological measurement is complicated, given that the simultaneous measurement of ECG, PPG, and BCG is feasible nowadays, researchers and developers are therefore recommended to decide on the sources of signal based on the target application. However, if the validation duration extended to multiple days, the significant decrease in predicting error suggests the influence of biorhythm [27].

The results of this paper are consistent with the results of Chowdhury et al. [6] by showing that Gaussian Process Regression can attain accurate BP estimation. Additionally, this research distinguishes itself by using the features that do not require the identification of the dicrotic notch and the diastolic peak and validating using a long-term dataset to highlight the necessity of the individual model.

This research emphasizes the importance of feature selection. There is no reason to replace the features which have been validated in previous studies with the automatically generated features using deep learning models in such a data-insufficient problem. At least, at the current stage, the domain knowledge is as important as the improvement of machine learning models.

This research shows the possibility of building an accurate BP estimation model by using the general features of PPG signal and HR. Moreover, given that the disappearance of diastolic peak is inevitable in aged people, who are the targeted population of the cuffless method, the general features could be a good alternative in modeling.

## 5. Conclusions

This paper, from the perspectives of data science and biomedical engineering, provides insightful results that are important for successive cuffless blood pressure studies. The main results of this research are (1) in the current stage of data insufficiency, an individual nonlinear regression model with intermittent calibration scheme is an ideal alternative for long-term use; (2) the general waveform features are used given the disappearance of morphological features caused by aging. Given the good fitting results of the nonlinear regression model, the general waveform features are informative in this regression problem and therefore can be used in the future studies.

## Figures and Tables

**Table 1:** Individual fitting results based on GPR_set1 model coupled with intermittent calibration.

Sub.	Ag	Gender	Ref. BP Range		MAE		RMSE	
No.	Yrs.	F/M	SBP	DBP	SBP	DBP	SBP	DBP
1	45	F	119–90	87–63	5.01	6.13	6.29	7.23
2	43	M	131–100	98–66	4.60	4.60	5.70	5.91
3	27	M	141–101	88–65	6.23	4.60	7.62	5.57
4	50	M	173–129	125–97	6.20	4.31	7.84	5.34
5	46	F	110–92	79–61	3.36	4.01	4.28	4.13
6	50	M	127–94	89–69	5.63	6.83	7.30	8.95
7	44	M	138–108	110–78	5.23	4.64	6.43	5.49
8	25	F	119–93	81–56	4.49	3.92	5.53	4.98
9	46	M	136–91	99–72	5.70	5.37	7.77	6.84
10	47	F	141–111	97–78	4.30	3.35	5.14	4.16
11	29	F	144–88	93–56	6.22	4.06	8.24	4.78

## Data Availability

All data analyzed during the current study are available from the corresponding author on reasonable request.
